# Associations of Education Attainment With Postpartum Depression and the Mediating Exploration: A Mendelian Randomization Study

**DOI:** 10.1155/da/8835118

**Published:** 2025-02-20

**Authors:** Xuanping Wang, Fang-Yue Zhou, Yanhui Hao, Jiaying Wu, Kaizhen Su, Si-Yue Chen, Wen Yu, Chen Zhang, Yan-Ting Wu, He-Feng Huang

**Affiliations:** ^1^The International Peace Maternity and Child Health Hospital, School of Medicine, Shanghai Jiao Tong University, Shanghai, China; ^2^Obstetrics and Gynecology Hospital, Institute of Reproduction and Development, Fudan University, Shanghai, China; ^3^Key Laboratory of Reproductive Genetics (Ministry of Education), Department of Reproductive Endocrinology, Women's Hospital, Zhejiang University School of Medicine, Hangzhou, China; ^4^Shanghai Key Laboratory of Reproduction and Development, Shanghai, China; ^5^Research Units of Embryo Original Diseases, Chinese Academy of Medical Sciences, Shanghai (No. 2019RU056), China

**Keywords:** childbearing age, education attainment, mediation analyses, mendelian randomization, postpartum depression

## Abstract

**Background:** Many studies have explored the relationship between education and postpartum depression (PPD), with inconsistent results. Our study is to identify which education-related factors (education attainment, qualifications, cognitive performance) played the predominant role in PPD using Mendelian randomization (MR) analysis. Then, we explored the factors that may mediate the effect of education on PPD.

**Method:** We performed two-sample multivariable Mendelian randomization (MVMR) to assess the independent impact of education-related factors on PPD. Based on the literature review, three mediating factors that may play a role in the path of education attainment and PPD were involved in mediation analysis, including childbearing age, neuroticism score, and average total household income before tax. Then, we used two-step MR and MVMR to estimate the indirect effect of these mediators.

**Results:** We identified genetically predicted 1-SD (3.71 years) higher education attainment (OR: 0.632; [95% confidential interval (CI): 0.464–0.860]); qualifications (OR: 0.418; [95% CI: 0.245–0.714]); or cognitive performance (OR: 0.770; [95% CI: 0.652–0.909]) was associated with lower risk of PPD, and the causal effects of education attainment (OR: 0.407; [95% CI: 0.214–0.773]) on PPD were independent of qualifications and cognition. Childbearing age (*β*: −0.497; [95% CI: −0.788−0.238]; *p*  < 0.001) and neuroticism score (*β*: −0.07; [95% CI: −0.120−0.030]; *p*  < 0.001) were identified as mediators of the association between education attainment and PPD.

**Conclusions:** These results suggested the predominant impact of education attainment on PPD independent of qualifications and cognition. Education level mainly affects PPD by changing the childbearing age.

**Trial Registration:** Chinese Clinical Trial Registry identifier: ChiCTR2000033433

## 1. Introduction

Postpartum depression (PPD), the onset of depressive symptoms after childbirth, develops at a critical period in women's life and can start at any time within the first year after childbirth, whose high-risk moment is located first 6 months after delivery [1]. PPD affects approximately 10% of women and has adverse implications for them and their infants [2]. According to the largest meta-analysis of PPD to date, the global prevalence of PPD was estimated to be ~17.22% [3]. PPD includes the typical symptoms of a series of depressive episodes, plus suicidal ideation and irrational fears for the child [4]. Beyond the suffering experienced by women during the postpartum period, maternal depression is also associated with infant behavioral problems, childhood depression, anxiety, poor school performance, cognitive development, and insecure attainment [5–7]. Hence, maternal depression can negatively impact the mother, the child's development, and the well-being of the entire family.

Many observational studies have shown that a lower education level was a risk factor for PPD [8]. A retrospective study also found that university education was an important protective factor against PPD [9]. People with higher education levels often perform better in cognitive function than those with lower education levels [10]. Moreover, there is a close connection between cognitive level and PPD. Mothers with PPD have been reported to have significant changes in metacognitive ability and are more likely to suffer from alexithymia [11]. In conclusion, factors such as educational background and cognition are closely related and connected to PPD [12, 13]. However, it remains unknown whether education attainment, qualifications, or cognitive performance has an independent causal effect on PPD.

It is noteworthy that maternal age is also associated with depression. The prevalence of PPD among adolescent mothers is twice as high as that among adult mothers [14]. Previous Mendelian randomization (MR) studies have shown strong associations between women's age at birth (AFB) and major depressive disorder (MDD) [15]. A household survey observed that women were more likely to have an early pregnancy experience if their mother had early childbearing experience. However, this association disappeared after adjusting for education [16]. Due to the interference of confounding factors, we are not sure whether childbearing age is an intermediate link between education and PPD. In addition, neurotic personality traits are more common in women with PPD than in healthy women [17]. Neurotic individuals lack emotion regulation strategies [18]. The peripartum period is thought to be a particularly vulnerable period for the manifestation of emotional lability. Socioeconomic status is also closely related to education level and PPD. High education and income were associated with higher rates of mental health service use, thus, reducing the risk of mental illness [19–21]. Therefore, we planned to explore the role of childbearing age, neuroticism, and household income between education attainment and PPD.

More and more studies implement the method of MR analysis using genetic variants as instrumental variables (IVs) for risk factors to test the causal link, which is less likely to be influenced by confounding and reverse causation [22–24]. Since genetic variations are randomly assigned during meiosis and fertilization, they are relatively independent of self-selective behaviors and are well-established long before the occurrence of diseases, thus minimizing the problems of confounding and reverse causation [22]. Because single nucleotide polymorphisms (SNPs) always precede the onset of disease, MR analysis could eliminate reverse causality and obtain a more reliable association than traditional observational studies [25]. In addition, the availability of GWAS data promotes the utilization of summary data-based MR. Multivariable MR is an extension of univariable MR that takes multiple genetic variants associated with several measured risk factors simultaneously into account [26]. Multivariable Mendelian randomization (MVMR) analysis can investigate the independent effects of primary and secondary exposures on an outcome and provide a consistent estimator of the direct effect of the exposure [27]. Two-step MR can be used to improve causal inference in mediation analysis, which has specific advantages. The causal effect of the exposure on the outcome, the exposure on the mediator, and the mediator on the outcome can all be tested [28].

In this study, we investigated the independent causal associations of education attainment, qualifications, or cognitive performance with PPD using two-sample MR. Our study also provided a way of thinking about the mediating factors of education attainment affecting PPD.

## 2. Methods

### 2.1. Study Design

**Figure 1:**
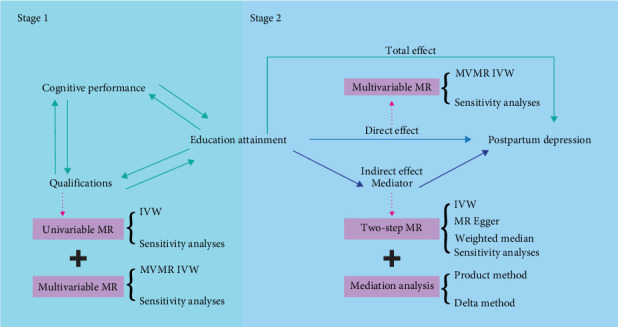
Study design. In stage 1, we assessed the causal associations of years of schooling, qualifications, and cognitive performance with PPD using UVMR and MVMR. The MVMR results further indicated that only years of schooling had an independent causal effect on PPD after adjusting for qualifications, cognitive performance, or both. In stage 2, we selected three mediators in the association between years of schooling and PPD and calculated their mediating effects using two-step MR and MVMR. IVW, inverse variance weighted; MVMR, multivariable Mendelian randomization; PPD, postpartum depression; UVMR, univariate Mendelian randomization.

This study included two stages of analysis (Figure 1). In stage 1, we assessed the causal associations of years of schooling, qualifications, and cognitive performance with PPD using univariate Mendelian randomization (UVMR) and MVMR, which used SNPs as IVs to proxy for each exposure. The MVMR results further indicated that only years of schooling had an independent causal effect on PPD after adjusting for qualifications, cognitive performance, or both. In stage 2, we selected three mediators in the association between education attainment and PPD and calculated their mediating effects using two-step MR and MVMR.

### 2.2. Data Sources

In our study, data were derived from GWASs conducted primarily in individuals of European ancestry (Table 1). All GWASs have received ethical approval from the relevant institutional review boards, participant informed consent, and stringent quality control. The main outcome trait GWAS (PPD) was from the FinnGen consortium [29] and exposures from consortiums, including SSGAC [30], Neale Lab, and Meta [31]. The sources of mediator traits GWAS were collected from MRC-IEU. Exposure and mediation data can be accessed through the OpenGWAS database API [32, 33].

#### 2.2.1. Exposures

Genetic instruments for education attainment were selected from a GWAS of years of schooling in 182,286 individuals of European ancestry (female-only data) conducted by the Social Science Genetic Association Consortium. Education attainment was derived by mapping the International Standard Classification of Education (ISCED) categories which was the ISCED of UNESCO in 1997. It includes a total of seven levels (0–6). Genetic instruments for qualifications were selected from a GWAS which included 334,070 samples (106,305 cases and 227,765 controls) of European ancestry. IVs for cognitive performance were selected from a GWAS meta-analysis which combined summary statistics from a published study of general cognitive ability in European-ancestry individuals (*N* = 35,298) conducted by the COGENT consortium with a new genome-wide association analysis of cognitive performance in the UK Biobank (*N* = 222,543). Final analyses were based on a sample-size weighted meta-analysis of these two results files (*N* = 257,841) [31].

#### 2.2.2. Outcomes

FinnGen research project is a public–private partnership combining genotype data from Finnish biobanks and digital health record data from Finnish health registries. We extracted the genetic associations of IVs with PPD from the FinnGen consortium, including 249,835 individuals using the International Classification of Diseases diagnosis codes of version 10. Among them, 236,178 individuals (female-only data) were without PPD, and 13,657 had PPD.

#### 2.2.3. Mediators

Based on the literature review, we selected three mediators that may lie on the pathways from education attainment to PPD with available genetic instruments derived from GWASs. The data sources of mediator GWAS were from UK Biobank, including age at first live birth (170,498 individuals), neuroticism score (374,323 individuals), and average total household income before tax (397,751 individuals). Household income was collected through a touchscreen questionnaire. The participants can choose an option that suits their situation from five options (less than £1800 to greater than £100,000).

### 2.3. Selection of Genetic Instruments

We applied stringent criteria to select effective SNPs as the genetic instruments from the GWAS summary data of all exposures. SNPs were selected with genome-wide significance (*p* < 5 × 10^−8^) of exposures and the clumping process (*R*^2^ > 0.001, window size = 10,000 kb) was executed to ensure that all the SNPs were not in linkage disequilibrium (LD). SNPs were excluded which were associated with the confounding factor of the outcome by using the PhenoScanner tool (http://www.phenoscanner.medschl.cam.ac.uk/). Proxy SNPs matched in summary data of the outcomes were not in high LD (*r*^2^ > 0.8).

## 3. Statistical Analyses

### 3.1. MR

MR analysis permits causal inferences about the link between the exposure and the outcome but needs to satisfy three core assumptions: (1) variants have to be reliably associated with exposure; (2) genetic variants must not be associated with confounders of the associations between instruments of each exposure and outcome; and (3) the effects of genetic variants on outcome must go through each exposure without horizontal pleiotropy [23].

Two-sample MR refers to the combination of summary statistics from GWAS of exposures in one population with summary statistics from GWAS of the outcome in another independent population [34]. We performed two-sample UVMR and MVMR to estimate the total and direct effects of each exposure on PPD. Inverse-variance weighted (IVW) was chosen as the primary method of MR results, which was an average of the Wald ratios where the weight of the SNP contribution was the inverse of the SNP effect on the outcome. Effect sizes were reported as odds ratio (OR) for binary outcome, *β* coefficient with corresponding 95% confidential interval (CI). All MR analyses were conducted using R packages“TwoSampleMR,” “MRPRESSO,” “MendelianRandomization [35],” “RMediation [36],”and “MVMR [37]” in R software (version 4.2.2; the R Foundation for Statistical Computing, Vienna, Austria).

### 3.2. Mediation MR Analyses

Mediation analysis is an approach to overcome some of the previous assumptions required for causal inference in mediation analysis [28]. Two-step MR is akin to the product of coefficient methods to assess the indirect effect by multiplying two estimates which are calculated from two steps including the causal effect of the exposure on the mediator (step 1) and the causal effect of the mediator on the outcome (step 2) [38]. In MVMR, the direct effect of the exposure on the outcome is estimated after controlling for each mediator [39].

We conducted a two-step MR to assess whether each intermediate risk factor had a mediating effect between years of schooling and PPD. The first step was to estimate the causal effect of genetically determined years of schooling on the mediator (*β*1) using UVMR, and in the second step, the causal effect of the mediator on PPD was estimated using MVMR with GWAS data from FinnGen (*β*2). We used the product method to calculate the indirect effect of each mediator by multiplying the results from the two steps (*β*1 × *β*2) and the 95% CIs were calculated based on SE estimated using the delta method [40–42].

### 3.3. MR Sensitivity Analyses

Alongside IVW, we conducted other complementary MR methods to validate the robustness of the causal estimates, including weighted median, MR Egger, MR pleiotropy residual sum, and Mendelian Randomization Pleiotropy RESidual Sum and Outlier (MR-PRESSO) in UVMR analyses. We applied the MVMR Egger method to validate the robustness of the IVW results in MVMR analyses. The weighted median method can provide valid SNP-specific estimates under the condition that >50% of the information contributing to the analysis comes from valid IVs [43]. The MR Egger regression method can assess whether directional pleiotropic effects of genetic variants on the outcome exist and provide valid estimates of the causal effect when the InSIDE (instrument strength independent of direct effect) assumption holds [44]. MR-PRESSO was also used to test and correct for potential horizontal pleiotropic outliers [45]. Cochran's *Q* test is an indicator of heterogeneity. When the *Q*-statistic for heterogeneity is high with a significant *p*-value, this suggests the presence of pleiotropy. In MVMR analyses, conditional *F*-statistics were applied to evaluate the instrument strength, with *F* >10 indicating suitable strength for the analysis [27].

## 4. Result

### 4.1. Total and Direct Effects of Years of Schooling, Cognitive Performance, and Qualifications on PPD

In the UVMR analysis, genetically predicted each 1-SD longer (3.7 years) years of schooling (OR: 0.632; [95% CI: 0.464–0.860], *p*: 0.004), higher qualifications (OR: 0.418; [95% CI: 0.245–0.714], *p* ≤ 0.001), and cognitive performance (OR: 0.770; [95% CI: 0.652–0.909], *p*: 0.002) were associated with a lower risk of PPD (Table S1). There were strong bidirectional causal associations between years of schooling, cognitive performance, and qualifications (Table S2).

For the heterogeneity test and pleiotropy test (MR Egger), all exposures showed no pleiotropic effects with all the *p*-values of Egger intercept being >0.05. There was no heterogeneity in years of schooling, while cognitive performance and qualifications showed heterogeneity (Table S3).

**Figure 2:**
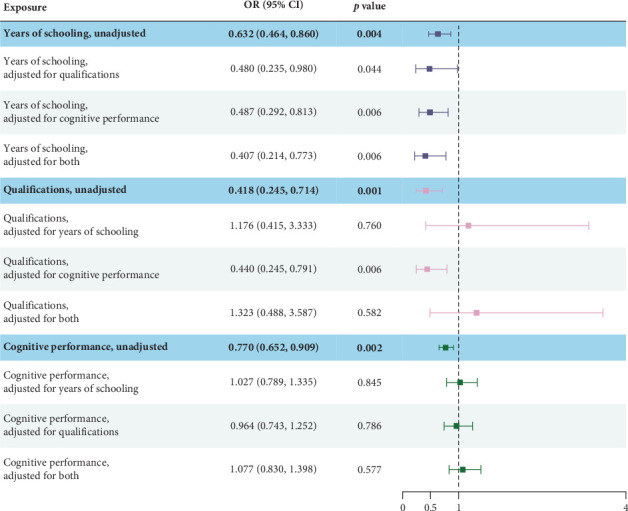
UVMR and MVMR estimates of the causal associations of education attainment, qualifications, and cognitive performance with PPD. UVMR and MVMR estimates of the causal associations of years of schooling, qualifications, and cognitive performance with PPD. Plots represent OR (95% CI). OR indicates the odds ratio. The bold words represent the result of univariate Mendel randomization. The relationship between each exposure and outcome and the adjusted results were represented by three different colors. MVMR, multivariable Mendelian randomization; PPD, postpartum depression; UVMR, univariate Mendelian randomization.

In MVMR analyses, the causal association between years of schooling and PPD remained after adjusting for cognitive performance (OR: 0.487; [95% CI: 0.292–0.813], *p*: 0.006), qualifications (OR: 0.480; [95% CI: 0.235–0.980], *p*: 0.044), or both of those factors (OR: 0.407; [95% CI: 0.214–0.773], *p*: 0.006), while the causal associations of qualifications (OR: 1.176; [95% CI: 0.415–3.333], *p*: 0.760) and cognitive performance (OR: 1.027; [95% CI: 0.789–1.335], *p*: 0.845) with PPD were no longer statistically significant after adjusting for years of schooling (Figure 2).

The *Q*-statistic for instrument validity varied from 201.75 to 367.51 and all genetic instruments of exposures showed persistent heterogeneity. Most of the statistical significance of IVW results in MVMR were consistent with those of MVMR Egger sensitivity analyses results, suggesting a low risk of bias due to horizontal pleiotropy (Table S4).

### 4.2. Two-Step MR to Test for Mediation

**Figure 3:**
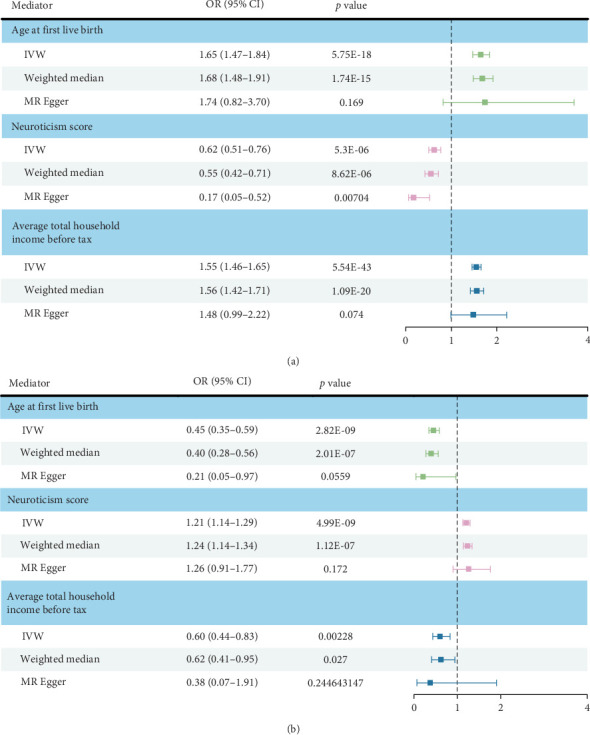
Two-step MR results evaluating potential mediators. (A) Step 1: Plots showing the effect of education attainment on each mediator (univariable MR). The effect was measured as the standard deviation change in mediator per education attainment change. (B) Step 2: Plots showing the odds of PPD per SD higher mediators (univariable MR). Bars indicate 95% confidence intervals around the point estimates from IVW, weighted median, and MR Egger analyses (in step 1: effect size/beta, in step 2: odds ratio). The presented data are available in Table S5 and Table S7. IVW, inverse-variance weighted; MR, Mendelian randomization; PPD, postpartum depression.

A two-step MR was performed to assess whether factors had a mediating effect between education attainment and PPD. In step 1, years of schooling were treated as the exposure and each mediator trait as the outcome; in step 2, the mediator was used as the exposure and PPD as the outcome. The results obtained by the IVW are presented in Figure 3.

In step 1, each 1-SD longer years of schooling was associated with higher age at first live birth (*β*: 0.498; [95% CI: 0.385 to 0.611], *p* < 0.001), lower neuroticism score (*β*: −0.475; [95% CI: −0.679 to −0.270], *p* < 0.001), higher average total household income before tax (*β*: 0.439; [95% CI: 0.376–0.502], *p* < 0.001). No pleiotropic effect was found in the association between each mediator and years of schooling except neuroticism score (Table S5). Heterogeneity was found in the association of years of schooling and age at first live birth (Table S6). We used *F*-statistics to test instrument validity (*F* >10)and all exposures had high instrument validity. In bidirectional MR analyses, there was little evidence that mediators decreased or increased education significantly (Table S9).

In step 2, higher age at first live birth (OR: 0.452; [95% CI: 0.348–0.588], *p* < 0.001), average total household income before tax (OR: 0.603; [95% CI: 0.435–0.834], *p*: 0.002) had the causal relationship with lower risk of PPD, while higher neuroticism score (OR: 1.211; [95% CI: 1.136–1.291], *p* < 0.001) was associated with higher risk of PPD. Genetic instruments of those mediators showed no pleiotropy with outcome, and at least 2 or 3 sensitivity analyses confirmed these IVW estimates had the same direction and statistical significance (Table S7). Heterogeneity in the association of each mediator and PPD is shown in Table S8.

### 4.3. Multivariable MR to Test for Mediation

MVMR analyses allowed us to establish the direct effect of education attainment after accounting for each mediator. The direct effects estimated are available in Table S10.

Pooled MVMR results showed that the direction and size of the effect of education attainment (*β*: 0.191; [95% CI: −0.406 to 0.788], *p*: 0.531) on PPD had undergone a subtle change after accounting for age at first live birth compared with the total effect from univariable MR. The protective effect of education attainment (*β*: −0.453; [95% CI: −0.785 to −0.121], *p* < 0.001) on PPD remained after adjusting for neuroticism score, while the significant effect of education attainment (*β*: −0.335; [95% CI: −0.989–0.318], *p*: 0.314) on the outcome disappeared after accounting for average total household income before tax. For the indirect effect of each mediator, except for the average total household income before tax effect (*β*: −0.059; [95% CI: −0.332–0.211], *p*: 0.669), the significant mediating effects of other mediating variables have remained.

### 4.4. Mediating Effects

Then, we carried out the mediation analysis to estimate the indirect effect of education attainment on PPD via selected mediators. The indirect effect (*β*1 × *β*2) was estimated as the product of coefficients of the effect of genetically determined education on the mediator (step 1 of two-step MR) and the effect of the mediator on the outcome with adjustment for education using MVMR. We performed the delta method to estimate the standard errors (SEs) and CI of the indirect effect (Table 2). Package “RMediation”provides functions to compute confidence intervals for a well-defined nonlinear function of the model parameters (e.g., product of *k* coefficients) in single-level and multilevel structural equation models.

Mediation analysis indicated that age at first live birth (*β*: −0.497; [95% CI: −0.788 to −0.238], *p* < 0.001) and neuroticism score (*β*: −0.07; [95% CI: −0.120 to −0.030], *p* < 0.001) mediated the association between years of schooling and PPD, while average total household income before tax (*β*: −0.059; [95% CI: −0.332 to 0.211], *p*: 0.669) did not strongly contribute to the protective effect of education attainment on PPD individually. Overall, the childbearing age played a complete mediating role in the association between education attainment and PPD, while the neuroticism score showed a partial mediating effect.

## 5. Discussion

For the first time, we identified education attainment as an independent protective contributor to PPD. The UVMR analysis showed that education attainment, cognitive performance, and qualifications all had a causal association with the relief of PPD. However, the causal impacts of cognitive performance and qualifications on PPD did not persist after adjustment for education attainment in the MVMR analysis, indicating that their effects were mainly influenced by education attainment. Next, we examined the potential mediators in the pathway from education attainment to PPD. We finally found that age at first live birth and neuroticism score lay on the pathways from education attainment to PPD, and education attainment appeared to exert the protective effect on PPD mainly by age at first live birth according to the results of MVMR.

Much evidence from observational studies had shown that higher educational attainment was a protective factor for PPD. A previous prospective study reported that women who had completed 15 years of education or more had a significantly reduced risk of postpartum depressive symptoms compared with those who had less than 13 years of education after adjustment for confounding factors [46]. Low cognitive levels can also lead to adverse psychosocial outcomes [47]. Moreover, educational level also affects people's access to social psychological services, which can lead to the development of anxiety disorders or depression. A national cohort study pointed out that those without any academic or professional qualifications have lower accessibility to psychological treatment [48]. Our analysis extended previous studies by differing three factors' total causal effects on PPD. A prospective cohort study observed significant decreases in self-efficacy and pregnancy-related nausea when women were pregnant, which indicated emotional regulation during pregnancy was in a state of vulnerability and fluctuation [49] and women's emotions were more easily affected by external adverse factors including discrimination during pregnancy and concerns about economic conditions. The educational level of mothers can play a certain role in flexible adjustment in the process from facing negative emotions to developing PPD. Some prospective data suggested that discrimination in pregnancy was prospectively associated with higher odds of PPD among women with low education after adjusting for other factors, including sociodemographic background. In contrast, perceived discrimination was not linked to PPD for women with high education [50]. The impact of perceived discrimination reported in pregnancy on PPD varies by education level. Our findings shed light on prioritizing education policies as powerful precautions against PPD and poor fetal birth outcomes.

Growing evidence points to the relationship between patterns of childbearing and maternal mental health status. Spence [51] found that early childbearing was linked with greater levels of depressive symptomatology in mature women. Birth cohort study [52] also highlighted that young mothers, particularly teenage mothers, were a vulnerable group with a high risk of poor mental health compared to mothers aged 25 years and above. Our study suggested that education had a profound impact on PPD through early child-bearing age. Highly educated women are more likely to avoid early childbearing, this is mainly because women with higher levels of education learn more fertility knowledge [53] and have the ability to plan their fertility progress. To be noted, when it comes to age as a risk factor, the conclusions are controversial in different literature. Some studies suggested that not giving birth to the first child and an older age at marriage were associated with higher depression scores [54]. Some studies discovered a marginally higher PPD prevalence in adolescent mothers than in adult mothers. Differences in cultural traditions [1] and the scale used when comparisons were made between countries with different socioeconomic structures may play a role in this disparity. These findings emphasize that further investigations are needed to clarify the mechanisms linking childbearing age to psychological well-being.

Neuroticism is defined as a temperamental sensitivity to negative stimulation and represents the tendency to experience various negative emotions, such as anxiety and anger, as well as the inability to relieve and cope with stress [55]. In addition, previous literature pointed out that neuroticism may increase stress perception, thereby increasing the production of pro-inflammatory messenger molecules and participating in the development of depression [56]. Prior research reported that even after adjusting for education, neuroticism was strongly associated with depressive symptoms in multiple regression models [57]. Our study suggested that the protective effect of education attainment on PPD can be achieved by reducing neuroticism scores, this was in line with previous research.

Current findings revealed that income inequalities were closely linked to PPD epidemiology and evidence showed that developing countries had a higher prevalence of PPD [3]. Our study observed that the total household income was negatively related to the risk of PPD. In today's rapidly updated and fiercely competitive society, education attainment is viewed as a sign of highly skilled labor. Our study indicated that higher educational backgrounds were the indicator of higher wages. That is mainly because higher education attainment means that individuals have higher skills, which translates into higher salaries [58]. One Migrants Dynamic Survey suggested that education level may increase the individual perception of happiness by increasing their economic income [21]. However, our study could not conclude that household income was an intermediary variable in the path of education level and PPD. A cross-sectional study from Japan showed that individuals with higher education attainment had lower depressive symptoms independently of household income level and individuals with lower household income levels had higher depressive symptoms independently of education level [59]. In addition, based on the review of previous literature, low household income as a positive factor for PPD has not been consistently verified. A prospective study observed that there were no relationships between household income or maternal educational levels and PPD [60]. It is worth noting that even among nations with similar economic strata, there are differences in the prevalence of PPD [3].

Our work has several strengths. First, the present study had methodological strengths. This was the first MR study to show the causal effects of education attainment on PPD independently of cognitive performance and qualifications, and we elucidated causal mediators in the pathway between the education level and PPD. Our study supported that education attainment reduced the risk of PPD mainly by increasing the childbearing age. Second, the high *F*-statistic (>10) of the genetic instruments involved in the UVMR analyses of exposures implied a lower chance of weak instrument bias. Although the genetic instruments employed in the MVMR analyses of household income and education level were relatively weak. The causality inferred for childbearing age and neuroticism score on depression were unlikely to be false positive due to both of these factors having appropriate IV strength. The robustness of the IVW estimates of our study was also supported by multiple MR sensitivity analyses. Third, we limited all the included participants to European ancestors, which allowed us to minimize the effect of population stratification.

This study also has some limitations. First, we identified the horizontal pleiotropy by adopting MR Egger intercept and MR-PRESSO tests and the information available in PhenoScanner. However, it was possible that the genetic instruments of exposure may influence the outcome via a currently unknown confounding factor. Second, the existence of heterogeneity of SNPs in MVMR analysis and inconsistent population gender (not limited to women only) from different GWASs may cause potential bias. Third, because all the participants involved in the analysis were European individuals, our results may not be directly generalizable to other ethnic groups.

In conclusion, our study found the causal protective impact of education attainment on the risk of PPD independently of qualifications and cognitive performance. We also discovered that education level reduced the risk of PPD mainly by the higher age at first live birth and neuroticism also played a minor role, but the mediating effect of household income between education attainment and PPD was not observed. Hence, the potential complex mechanisms between education and PPD may need further investigations.

## Figures and Tables

**Table 1:** Summary of the GWAS data used in the MR analyses.

Phenotype	Unit	Participants	Ancestry	Consortium	Sex	PMID and/or data source
PPD	Event	249,835	European	FinnGen (2022)	Females	NA/https://r8.finngen.fi/pheno/O15_POSTPART_DEPR
Years of schooling	SD (3.71 years)	182,286	European	SSGAC (2016)	Females	27225129/https://gwas.mrcieu.ac.uk/datasets/ieu-a-1011/
Qualifications	SD	334,070	European	UK Biobank (2017)	Males and females	NA/http://www.nealelab.is/uk-biobank
Cognitive performance	SD	257,841	European	Meta (2018)	Males and females	30038396/https://www.ebi.ac.uk/gwas/publications/30038396
Age at first live birth	SD	170,498	European	UK Biobank (2018)	Females	NA/http://www.nealelab.is/uk-biobank
Neuroticism score	SD	374,323	European	UK Biobank (2018)	Males and females	NA/https://gwas.mrcieu.ac.uk/datasets/ukb-b-4630/
Average total household income before tax	SD	397,751	European	UK Biobank (2018)	Males and females	29846171/https://gwas.mrcieu.ac.uk/datasets/ukb-b-7408/

Abbreviations: GWAS, genome-wide association study; MR, Mendelian randomization; NA, not available; PPD, postpartum depression; SSGAC, Social Science Genetic Association Consortium.

**Table 2:** Mediation analysis results.

Mediator	*β*1	se_*β*1	*β*2	se_*β*2	*β*1 × *β*2 (95% CI)	se_*β*1 × *β*2	Mediation effect, *p* value
Age at first live birth	0.498	0.058	−0.998	0.255	−0.497 (−0.788 to −0.238)	0.140	3.80E-04
Neuroticism score	−0.475	0.104	0.147	0.036	−0.070 (−0.120 to −0.030)	0.023	2.30E-03
Average total household income before tax	0.439	0.032	−0.135	0.314	−0.059 (−0.332 to 0.211)	0.138	0.669

*Note:* The indirect effect on the outcome via each mediator and the significance test of the mediating effect. The indirect effect was calculated as the product of coefficients of the total effect of education attainment on each mediator (step 1 of two-step MR) and the direct effect of each mediator on PPD (MVMR), that is, product method, and the 95% CIs were shown based on SE estimated using delta method.

## Data Availability

All data used in the present study were available from genome-wide association study summary statistics that were publicly released. The GWAS datasets used in this study are available in OpenGWAS (https://gwas.mrcieu.ac.uk/datasets/) and FinnGen study (https://r8.finngen.fi/pheno/O15_POSTPART_DEPR).

## Supporting Information

Supporting Information Table S1. UVMR estimating the association of years of schooling, qualifications, cognitive performance with PPD. We found that longer years of education, higher qualifications, and cognitive performance were associated with lower PPD risk. Table S2. UVMR estimating the bidirectional association between years of schooling, qualifications, or cognitive performance. There were strong bidirectional causal associations between years of schooling, cognitive performance, and qualifications. Table S3. MR heterogeneity test and pleiotropy test (MR Egger) of the association of years of schooling, qualifications, and cognitive performance with PPD. There was no heterogeneity in years of schooling, while cognitive performance and qualifications showed heterogeneity. Table S4. MVMR estimating the association of years of schooling, qualifications, and cognitive performance with PPD. Table S4 is a supplement to Figure 2. The *Q*-statistic for instrument validity varied from 201.75 to 367.51 and all genetic IV of exposures showed persistent heterogeneity. Most of the statistical significance of IVW results in MVMR were consistent with those of MVMR Egger sensitivity analyses results, suggesting a low risk of bias due to horizontal pleiotropy. Table S5. UVMR estimating the association between years of schooling and each mediator. Table S5 is a supplement to Figure 3A. No pleiotropic effect was found in the association between each mediator and years of schooling except neuroticism score. Table S6. MR heterogeneity test of the association of years of schooling with each mediator. Table S6 is also a supplement to Figure 3A. Heterogeneity was found in the association of years of schooling and age at first live birth. Table S7. UVMR estimating the association between each mediator and PPD. Table S7 is a supplement to Figure 3B. Genetic IVs of those mediators showed no pleiotropy with outcome, and at least 2 or 3 sensitivity analyses confirmed these IVW estimates had the same direction and statistical significance. Table S8. MR heterogeneity test of the association of each mediator and PPD. Heterogeneity in the association of each mediator and PPD is shown in Table S8 which is a supplement to Figure 3B. Table S9. Reverse MR estimating the association of each mediator with education attainment. According to the results of the IVW method, we found that there was no reverse causal relationship between the mediating factor and education attainment. Table S10. MVMR estimating the association of each mediator with PPD with adjustment for education attainment. MVMR analyses allowed us to establish the direct effect of education attainment after accounting for each mediator. The direct effects estimated are available in Table S10.

## References

[1] Evagorou O., Arvaniti A., Samakouri M. (2016). Cross-Cultural Approach of Postpartum Depression: Manifestation, Practices Applied, Risk Factors and Therapeutic Interventions. *Psychiatric Quarterly*.

[2] Ko J. Y., Rockhill K. M., Tong V. T., Morrow B., Farr S. L. (2017). Trends in Postpartum Depressive Symptoms —27 States, 2004, 2008, and 2012. *MMWR. Morbidity and Mortality Weekly Report*.

[3] Wang Z., Liu J., Shuai H. (2021). Mapping Global Prevalence of Depression Among Postpartum Women. *Translational Psychiatry*.

[4] Howard L. M., Flach C., Mehay A., Sharp D., Tylee A. (2011). The Prevalence of Suicidal Ideation Identified by the Edinburgh Postnatal Depression Scale in Postpartum Women in Primary Care: Findings From the RESPOND Trial. *BMC Pregnancy and Childbirth*.

[5] Bödeker K., Fuchs A., Führer D. (2019). Impact of Maternal Early Life Maltreatment and Maternal History of Depression on Child Psychopathology: Mediating Role of Maternal Sensitivity?. *Child Psychiatry & Human Development*.

[6] Letourneau N., Dewey D., Kaplan B. J. (2019). Intergenerational Transmission of Adverse Childhood Experiences via Maternal Depression and Anxiety and Moderation by Child Sex. *Journal of Developmental Origins of Health and Disease*.

[7] Schickedanz A., Halfon N., Sastry N., Chung P. J. (2018). Parents’ Adverse Childhood Experiences and Their Children’s Behavioral Health Problems. *Pediatrics*.

[8] Matsumura K., Hamazaki K., Tsuchida A., Kasamatsu H., Inadera H., Japan Environment and Children’s Study (JECS) Group (2019). Education Level and Risk of Postpartum Depression: Results From the Japan Environment and Children’s Study (JECS). *BMC Psychiatry*.

[9] Grussu P., Quatraro R. M. (2009). Prevalence and Risk Factors for a High Level of Postnatal Depression Symptomatology in Italian Women: A Sample Drawn From Ante-Natal Classes. *European Psychiatry*.

[10] Lenehan M. E., Summers M. J., Saunders N. L., Summers J. J., Vickers J. C. (2015). Relationship Between Education and Age-Related Cognitive Decline: A Review of Recent Research. *Psychogeriatrics*.

[11] Diop S., Turmes L., Specht C., Seehagen S., Juckel G., Mavrogiorgou P. (2022). Capacities for Meta-Cognition, Social Cognition, and Alexithymia in Postpartum Depression. *Psychiatry Research*.

[12] Donati G., Dumontheil I., Meaburn E. L. (2019). Genome-Wide Association Study of Latent Cognitive Measures in Adolescence: Genetic Overlap With Intelligence and Education. *Mind, Brain, and Education*.

[13] Savage J. E., Jansen P. R., Stringer S. (2018). Genome-Wide Association Meta-Analysis in 269,867 Individuals Identifies New Genetic and Functional Links to Intelligence. *Nature Genetics*.

[14] Dinwiddie K. J., Schillerstrom T. L., Schillerstrom J. E. (2017). Postpartum Depression in Adolescent Mothers. *Journal of Psychosomatic Obstetrics & Gynecology*.

[15] Wang Z., Lu J., Weng W., Fu J., Zhang J. (2023). Women’s Reproductive Traits and Major Depressive Disorder: A Two-Sample Mendelian Randomization Study. *Journal of Affective Disorders*.

[16] Almeida M. C. C., Aquino E. M. L. (2009). The Role of Education Level in the Intergenerational Pattern of Adolescent Pregnancy in Brazil. *International Perspectives on Sexual and Reproductive Health*.

[17] Maliszewska K., Bidzan M., Świątkowska-Freund M., Preis K. (2017). Medical and Psychosocial Determinants of Risk of Postpartum Depression: A Cross-Sectional Study. *Acta Neuropsychiatrica*.

[18] Paulus D. J., Vanwoerden S., Norton P. J., Sharp C. (2016). Emotion Dysregulation, Psychological Inflexibility, and Shame as Explanatory Factors Between Neuroticism and Depression. *Journal of Affective Disorders*.

[19] Borges G., Aguilar-Gaxiola S., Andrade L. (2020). Twelve-Month Mental Health Service Use in Six Countries of the Americas: A Regional Report From the World Mental Health Surveys. *Epidemiology and Psychiatric Sciences*.

[20] Veenstra G., Vanzella-Yang A. (2021). Does Household Income Mediate the Association Between Education and Health in Canada?. *Scandinavian Journal of Public Health*.

[21] Yang D., Zheng G., Wang H., Li M. (2022). Education, Income, and Happiness: Evidence From China. *Frontiers in Public Health*.

[22] Davies N. M., Holmes M. V., Davey Smith G. (2018). Reading Mendelian Randomisation Studies: A Guide, Glossary, and Checklist for Clinicians. *BMJ*.

[23] Lawlor D. A., Harbord R. M., Sterne J. A. C., Timpson N., Davey Smith G. (2008). Mendelian Randomization: Using Genes as Instruments for Making Causal Inferences in Epidemiology. *Statistics in Medicine*.

[24] Wang Y., Ye C., Kong L. (2023). Independent Associations of Education, Intelligence, and Cognition With Hypertension and the Mediating Effects of Cardiometabolic Risk Factors: A Mendelian Randomization Study. *Hypertension*.

[25] Zeng Z., Zhang W., Qian Y. (2020). Association of Telomere Length With Risk of Rheumatoid Arthritis: A Meta-Analysis and Mendelian Randomization. *Rheumatology*.

[26] Burgess S., Thompson S. G. (2015). Multivariable Mendelian Randomization: The Use of Pleiotropic Genetic Variants to Estimate Causal Effects. *American Journal of Epidemiology*.

[27] Sanderson E., Davey Smith G., Windmeijer F., Bowden J. (2019). An Examination of Multivariable Mendelian Randomization in the Single-Sample and Two-Sample Summary Data Settings. *International Journal of Epidemiology*.

[28] Carter A. R., Sanderson E., Hammerton G. (2021). Mendelian Randomisation for Mediation Analysis: Current Methods and Challenges for Implementation. *European Journal of Epidemiology*.

[29] Kurki M. I., Karjalainen J., Palta P. (2023). FinnGen Provides Genetic Insights From a Well-Phenotyped Isolated Population. *Nature*.

[30] Okbay A., Beauchamp J. P., Fontana M. A. (2016). Genome-Wide Association Study Identifies 74 Loci Associated With Educational Attainment. *Nature*.

[31] Lee J. J., Wedow R., Okbay A. (2018). Gene Discovery and Polygenic Prediction From a Genome-Wide Association Study of Educational Attainment in 1.1 Million Individuals. *Nature Genetics*.

[32] Elsworth B., Lyon M., Alexander T. (2020). *The MRC IEU OpenGWAS Data Infrastructure*.

[33] Hemani G., Zheng J., Elsworth B. (2018). The MR-Base Platform Supports Systematic Causal Inference Across the Human Phenome. *eLife*.

[34] Ference B. A., Holmes M. V., Smith G. D. (2021). Using Mendelian Randomization to Improve the Design of Randomized Trials. *Cold Spring Harbor Perspectives in Medicine*.

[35] Yavorska O. O., Burgess S. (2017). MendelianRandomization: An R Package for Performing Mendelian Randomization Analyses Using Summarized Data. *International Journal of Epidemiology*.

[36] Tofighi D. (2020). Bootstrap Model-Based Constrained Optimization Tests of Indirect Effects. *Frontiers in Psychology*.

[37] Sanderson E., Spiller W., Bowden J. (2021). Testing and Correcting for Weak and Pleiotropic Instruments in Two-Sample Multivariable Mendelian Randomization. *Statistics in Medicine*.

[38] Relton C. L., Davey Smith G. (2012). Two-Step Epigenetic Mendelian Randomization: A Strategy for Establishing the Causal Role of Epigenetic Processes in Pathways to Disease. *International Journal of Epidemiology*.

[39] Burgess S., Daniel R. M., Butterworth A. S., Thompson S. G., the EPIC-InterAct Consortium (2015). Network Mendelian Randomization: Using Genetic Variants as Instrumental Variables to Investigate Mediation in Causal Pathways. *International Journal of Epidemiology*.

[40] MacKinnon D. P., Fritz M. S., Williams J., Lockwood C. M. (2007). Distribution of the Product Confidence Limits for the Indirect Effect: Program PRODCLIN. *Behavior Research Methods*.

[41] Meeker W. Q., Escobar L. A. (1994). An Algorithm to Compute the Cdf of the Product of Two Normal Random Variables. *Communications in Statistics—Simulation and Computation*.

[42] Tofighi D., MacKinnon D. P. (2011). RMediation: An R Package for Mediation Analysis Confidence Intervals. *Behavior Research Methods*.

[43] Bowden J., Davey Smith G., Haycock P. C., Burgess S. (2016). Consistent Estimation in Mendelian Randomization With Some Invalid Instruments Using a Weighted Median Estimator. *Genetic Epidemiology*.

[44] Bowden J., Davey Smith G., Burgess S. (2015). Mendelian Randomization With Invalid Instruments: Effect Estimation and Bias Detection Through Egger Regression. *International Journal of Epidemiology*.

[45] Verbanck M., Chen C.-Y., Neale B., Do R. (2018). Detection of Widespread Horizontal Pleiotropy in Causal Relationships Inferred From Mendelian Randomization Between Complex Traits and Diseases. *Nature Genetics*.

[46] Miyake Y., Tanaka K., Arakawa M. (2020). Associations of Job Type, Income, and Education With Postpartum Depressive Symptoms: The Kyushu Okinawa Maternal and Child Health Study. *Psychiatry Research*.

[47] McIntyre R. S., Cha D. S., Soczynska J. K. (2013). Cognitive Deficits and Functional Outcomes in Major Depressive Disorder: Determinants, Substrates, and Treatment Interventions. *Depression and Anxiety*.

[48] Sharland E., Rzepnicka K., Schneider D. (2023). Socio-Demographic Differences in Access to Psychological Treatment Services: Evidence From a National Cohort Study. *Psychological Medicine*.

[49] Yu Z. M., Van Blyderveen S., Schmidt L. (2023). Do Psychological and Behavioural Factors Change Over Pregnancy?. *Journal of Obstetrics and Gynaecology Canada*.

[50] Stepanikova I., Kukla L. (2017). Is Perceived Discrimination in Pregnancy Prospectively Linked to Postpartum Depression? Exploring the Role of Education. *Maternal and Child Health Journal*.

[51] Spence N. J. (2008). The Long-Term Consequences of Childbearing: Physical and Psychological Well-Being of Mothers in Later Life. *Research on Aging*.

[52] Aitken Z., Hewitt B., Keogh L., LaMontagne A. D., Bentley R., Kavanagh A. M. (2016). Young Maternal Age at First Birth and Mental Health Later in Life: Does the Association Vary by Birth Cohort?. *Social Science & Medicine*.

[53] Swift B. E., Liu K. E. (2014). The Effect of Age, Ethnicity, and Level of Education on Fertility Awareness and Duration of Infertility. *Journal of Obstetrics and Gynaecology Canada*.

[54] Green K., Broome H., Mirabella J. (2006). Postnatal Depression Among Mothers in the United Arab Emirates: Socio-Cultural and Physical Factors. *Psychology, Health & Medicine*.

[55] Abbasi I. S. (2016). The Role of Neuroticism in the Maintenance of Chronic Baseline Stress Perception and Negative Affect. *The Spanish Journal of Psychology*.

[56] Schmidt F. M., Sander C., Minkwitz J. (2018). Serum Markers of Inflammation Mediate the Positive Association Between Neuroticism and Depression. *Frontiers in Psychiatry*.

[57] Jylhä P., Isometsä E. (2006). The Relationship of Neuroticism and Extraversion to Symptoms of Anxiety and Depression in the General Population. *Depression and Anxiety*.

[58] Weiss A. (1995). Human Capital vs. Signalling Explanations of Wages. *Journal of Economic Perspectives*.

[59] Hinata A., Kabasawa K., Watanabe Y. (2021). Education, Household Income, and Depressive Symptoms in Middle-Aged and Older Japanese Adults. *BMC Public Health*.

[60] Miyake Y., Tanaka K., Sasaki S., Hirota Y. (2011). Employment, Income, and Education and Risk of Postpartum Depression: The Osaka Maternal and Child Health Study. *Journal of Affective Disorders*.

