# Supplementary material for: Associations of Education Attainment With Postpartum Depression and the Mediating Exploration: A Mendelian Randomization Study
Source: Depress Anxiety. 2025 Feb 20;2025:8835118. doi: 10.1155/da/8835118 (PMC11919117; doi:10.1155/da/8835118)
Supplement: Supporting Information — Table S1. UVMR estimating the association of years of schooling, qualifications, cognitive performance with PPD. We found that longer years of education, higher qualifications, and cognitive performance were associated with lower PPD risk. Table S2. UVMR estimating the bidirectional association between years of schooling, qualifications, or cognitive performance. There were strong bidirectional causal associations between years of schooling, cognitive performance, and qualifications. Table S3. MR heterogeneity test and pleiotropy test (MR Egger) of the association of years of schooling, qualifications, and cognitive performance with PPD. There was no heterogeneity in years of schooling, while cognitive performance and qualifications showed heterogeneity. Table S4. MVMR estimating the association of years of schooling, qualifications, and cognitive performance with PPD. Table S4 is a supplement to Figure 2. The Q-statistic for instrument validity varied from 201.75 to 367.51 and all genetic IV of exposures showed persistent heterogeneity. Most of the statistical significance of IVW results in MVMR were consistent with those of MVMR Egger sensitivity analyses results, suggesting a low risk of bias due to horizontal pleiotropy. Table S5. UVMR estimating the association between years of schooling and each mediator. Table S5 is a supplement to Figure 3A. No pleiotropic effect was found in the association between each mediator and years of schooling except neuroticism score. Table S6. MR heterogeneity test of the association of years of schooling with each mediator. Table S6 is also a supplement to Figure 3A. Heterogeneity was found in the association of years of schooling and age at first live birth. Table S7. UVMR estimating the association between each mediator and PPD. Table S7 is a supplement to Figure 3B. Genetic IVs of those mediators showed no pleiotropy with outcome, and at least 2 or 3 sensitivity analyses confirmed these IVW estimates had the same direction and [file 8835118.f1.pdf]

Table S1. UVMR estimating the association of years of schooling, qualifications, cognitive performance with PPD

Exposure	Method	nSNPs	OR(95% CI)	P value
Years of schooling	IVW	18	0.632(0.464, 0.860)	0.004
	weighted median		0.714(0.467, 1.093)	0.121
	MR Egger		2.012(0.277, 14.619)	0.499
	Simple mode		0.622(0.305, 1.270)	0.210
	Weighted mode		0.693(0.342, 1.407)	0.325
Qualifications	IVW	45	0.418(0.245, 0.714)	0.001
	weighted median		0.495(0.247, 0.993)	0.048
	MR Egger		2.013(0.074, 55.121)	0.681
	Simple mode		0.614(0.127, 2.970)	0.547
	Weighted mode		0.631(0.179, 2.222)	0.478
Cognitive performance	IVW	132	0.770(0.652, 0.909)	0.002
	weighted median		0.830(0.681, 1.011)	0.065
	MR Egger		0.753(0.341, 1.662)	0.483
	Simple mode		0.904(0.521, 1.568)	0.720
	Weighted mode		0.919(0.530, 1.593)	0.763

Abbreviations: CI, confidence interval; IVW, inverse variance weighted; MR, Mendelian randomization; SNP, single nucleotide polymorphism; UVMR, univariable Mendelian randomization.

Table S2. UVMR estimating the bidirectional association between years of schooling, qualifications, or cognitive performance

Exposure	Outcome	Method	nSNPs	β(95% CI）	P value	PRESSO Global test P-value	Pleiotropy test P-value
Years of schooling	Qualifications	IVW	7	0.250(0.206, 0.294)	1.39E-28	0.935	0.470
		weighted median		0.236(0.167, 0.306)	3.07E-11		
		MR Egger		-0.002(-0.634, 0.631)	0.996		
	Cognitive performance	IVW	13	0.554(0.476, 0.632)	1.13E-43	0.969	0.847
		weighted median		0.544(0.426, 0.662)	1.41E-19		
		MR Egger		0.495(-0.096, 1.086)	0.129		
Qualifications	Years of schooling	IVW	42	1.381(1.271, 1.492)	4.45E-132	0.836	0.119
		weighted median		1.234(1.064, 1.404)	8.18E-46		
		MR Egger		0.624(-0.315, 1.562)	0.200		
	Cognitive performance	IVW	32	1.154(1.025, 1.283)	1.76E-68	0.353	0.115
		weighted median		1.062(0.884, 1.240)	1.61E-31		
		MR Egger		0.372(-0.581, 1.326)	0.450		
Cognitive performance	Years of schooling	IVW	129	0.352(0.312, 0.391)	4.31E-67	<0.001	0.861
		weighted median		0.311(0.260, 0.363)	7.16E-33		
		MR Egger		0.368(0.184, 0.551)	1.38E-04		
	Qualifications	IVW	106	0.203(0.188, 0.218)	4.03E-146	0.018	0.476
		weighted median		0.204(0.185, 0.223)	7.40E-95		
		MR Egger		0.231(0.153, 0.310)	8.34E-08		

Abbreviations: CI, confidence interval; IVW, inverse variance weighted; MR, Mendelian randomization; SNP, single nucleotide polymorphism; UVMR, univariable Mendelian randomization.

Table S3. MR heterogeneity test and pleiotropy test (MR Egger) of the association of years of schooling, qualifications, cognitive performance with PPD

Exposure	Method	Q-statistic	Q-df	Egger intercept	Pleiotropy p-value
Years of schooling	IVW	17.90	17	-0.027	0.26
	MR Egger	16.51	16		
Qualifications	IVW	68.56	44	-0.016	0.35
	MR Egger	67.17	43		
Cognitive performance	IVW	233.54	131	0.000	0.96
	MR Egger	233.53	130		

Abbreviations: df, degree of freedom; IVW, inverse variance weighted; MR, Mendelian randomization.

Table S4. MVMR estimating the association of years of schooling, qualifications and cognitive performance with PPD

<table>
<tr><th>Method</th><th>Exposure</th><th>β</th><th>SE</th><th>P</th><th colspan="3">MVMR
Instrument validity test</th><th colspan="2">MVMR
Heterogeneity test</th><th colspan="2">MVMR
Pleiotropy test</th></tr>
<tr><th colspan="5"></th><th>F-statistic</th><th>Q statistic</th><th>P value</th><th>Q statistic</th><th>P value</th><th>Egger intercept</th><th>P value</th></tr>
<tr><td rowspan="3">MV-IVW</td><td>Years of schooling</td><td>-0.90</td><td>0.33</td><td>0.01</td><td rowspan="6">10.38</td><td rowspan="6">330.25</td><td rowspan="6">2.23E-07</td><td rowspan="3">345.96</td><td rowspan="3">＜0.05</td><td rowspan="6">-0.001</td><td rowspan="6">0.86</td></tr>
<tr><td>Qualifications</td><td>0.28</td><td>0.51</td><td>0.58</td></tr>
<tr><td>Cognitive performance</td><td>0.07</td><td>0.13</td><td>0.58</td></tr>
<tr><td rowspan="3">MVMR Egger</td><td>Years of schooling</td><td>-0.90</td><td>0.33</td><td>0.01</td><td rowspan="3">345.91</td><td rowspan="3">＜0.05</td></tr>
<tr><td>Qualifications</td><td>0.32</td><td>0.56</td><td>0.57</td></tr>
<tr><td>Cognitive performance</td><td>0.10</td><td>0.19</td><td>0.61</td></tr>
<tr><td rowspan="2">MV-IVW</td><td>Years of schooling</td><td>-0.73</td><td>0.36</td><td>0.04</td><td rowspan="4">13.93</td><td rowspan="4">271.00</td><td rowspan="4">1.04E-06</td><td rowspan="2">279.29</td><td rowspan="2">＜0.05</td><td rowspan="4">-0.004</td><td rowspan="4">0.56</td></tr>
<tr><td>Qualifications</td><td>0.16</td><td>0.53</td><td>0.76</td></tr>
<tr><td rowspan="2">MVMR Egger</td><td>Years of schooling</td><td>-0.75</td><td>0.37</td><td>0.04</td><td rowspan="2">278.72</td><td rowspan="2">＜0.05</td></tr>
<tr><td>Qualifications</td><td>0.62</td><td>0.94</td><td>0.51</td></tr>
<tr><td rowspan="2">MV-IVW</td><td>Years of schooling</td><td>-0.72</td><td>0.26</td><td>0.01</td><td rowspan="4">9.61</td><td rowspan="4">201.75</td><td rowspan="4">7.08E-05</td><td rowspan="2">207.67</td><td rowspan="2">＜0.05</td><td rowspan="4">-0.006</td><td rowspan="4">0.42</td></tr>
<tr><td>Cognitive performance</td><td>0.03</td><td>0.13</td><td>0.85</td></tr>
<tr><td rowspan="2">MVMR Egger</td><td>Years of schooling</td><td>-0.73</td><td>0.26</td><td>0.01</td><td rowspan="2">206.64</td><td rowspan="2">＜0.05</td></tr>
<tr><td>Cognitive performance</td><td>0.32</td><td>0.39</td><td>0.41</td></tr>
<tr><td rowspan="2">MV-IVW</td><td>Qualifications</td><td>-0.82</td><td>0.30</td><td>0.01</td><td rowspan="4">11.51</td><td rowspan="4">367.51</td><td rowspan="4">2.46E-10</td><td rowspan="2">369.24</td><td rowspan="2">＜0.05</td><td rowspan="4">0.000</td><td rowspan="4">0.96</td></tr>
<tr><td>Cognitive performance</td><td>-0.04</td><td>0.13</td><td>0.79</td></tr>
<tr><td rowspan="2">MVMR Egger</td><td>Qualifications</td><td>-0.83</td><td>0.36</td><td>0.02</td><td rowspan="2">369.24</td><td rowspan="2">＜0.05</td></tr>
<tr><td>Cognitive performance</td><td>-0.04</td><td>0.19</td><td>0.82</td></tr>
</table>

Abbreviations: MV-IVW, multivariable inverse variance weighted; MVMR, multivariable Mendelian randomization; SE, standard error.

Table S5. UVMR estimating the association between years of schooling and each mediator

Mediator	Method	nSNPs	β(95% CI)	P value	Pleiotropy test	
					Egger intercept	P
Age at first live birth	IVW	19	0.498(0.385, 0.611)	5.75E-18	-0.001	0.887
	weighted median		0.521(0.392, 0.648)	1.74E-15		
	MR Egger		0.553(-0.202, 1.308)	0.169		
Neuroticism score	IVW	18	-0.475(-0.679, -0.270)	5.30E-06	0.030	0.035
	weighted median		-0.601(-0.866, -0.336)	8.62E-06		
	MR Egger		-1.782(-2.912, -0.651)	7.04E-03		
Average total household income before tax	IVW	17	0.439(0.376, 0.502)	5.54E-43	0.001	0.827
	weighted median		0.443(0.350, 0.537)	1.09E-20		
	MR Egger		0.394(-0.008, 0.796)	0.074		

Abbreviations: CI, confidence interval; IVW, inverse variance weighted; MR, Mendelian randomization; SNP, single nucleotide polymorphism; UVMR, univariable Mendelian randomization.

Table S6. MR heterogeneity test of the association of years of schooling with each mediator

Exposure	Mediator	Method	Q statistic	Q df	Q p-value
Years of schooling	Age at first live birth	IVW	40.732	18	0.002
		MR Egger	40.683	17	0.001
	Neuroticism score	IVW	25.894	17	0.076
		MR Egger	19.475	16	0.245
	Average total household income before tax	IVW	9.595	16	0.887
		MR Egger	9.546	15	0.847

Abbreviations: df, degree of freedom; IVW, inverse variance weighted; MR, Mendelian randomization.

Table S7. UVMR estimating the association between each mediator and PPD

Mediator	Method	nSNPs	OR(95% CI)	P value	Pleiotropy test	
					**Egger intercept**	**P**
Age at first live birth	IVW	28	0.452 (0.348, 0.588)	2.82E-09	0.017	0.330
	weighted median		0.397 (0.280, 0.562)	2.01E-07		
	MR Egger		0.212 (0.047, 0.968)	0.056		
Neuroticism score	IVW	108	1.211 (1.136, 1.291)	4.99E-09	-0.002	0.798
	weighted median		1.238 (1.144, 1.339)	1.12E-07		
	MR Egger		1.264 (0.905, 1.766)	0.172		
Average total household income before tax	IVW	40	0.603 (0.435, 0.834)	0.002	0.009	0.563
	weighted median		0.624 (0.411, 0.948)	0.027		
	MR Egger		0.375 (0.074, 1.907)	0.245		

Abbreviations: CI, confidence interval; IVW, inverse variance weighted; MR, Mendelian randomization; OR, odds ratio; SNP, single nucleotide polymorphism; UVMR, univariable Mendelian randomization.

Table S8. MR heterogeneity test of the association of each mediator and PPD

Outcome	Mediator	Method	Q statistic	Q df	Q p-value
PPD	Age at first live birth	IVW	32.073	27	0.229
		MR Egger	30.903	26	0.232
	Neuroticism score	IVW	152.84	107	0.002
		MR Egger	152.745	106	0.002
	Average total household income before tax	IVW	65.036	39	0.006
		MR Egger	64.459	38	0.005

Abbreviations: df, degree of freedom; IVW, inverse variance weighted; MR, Mendelian randomization.

Table S9. Reverse MR estimating the association of each mediator with education attainment

Mediator	Method	MR results			Heterogeneity test		Directional pleiotropy test	
		**β**	**SE**	**p-value**	**Q statistic**	**Q p-value**	**Egger intercept**	**P value**
Age at first live birth	IVW	0.388	0.043	0.17	63.71	1.34E-04	-0.0008	0.9037
	Weighted Median	0.352	0.049	5.00E-13	NA	NA		
	MR Egger	0.425	0.302	3.89E-19	63.67	8.54E-05		
Neuroticism score	IVW	-0.013	0.048	0.79	190.05	5.42E-07	-0.0024	0.3423
	Weighted Median	-0.048	0.011	1.17E-05	NA	NA		
	MR Egger	-0.057	0.009	4.43E-10	188.38	5.86E-07		
Average total household income before tax	IVW	0.261	0.194	0.19	62.16	0.00797724	0.0064	0.0841
	Weighted Median	0.553	0.051	2.97E-27	NA	NA		
	MR Egger	0.598	0.041	9.52E-49	57.29	0.01776386		

Abbreviations: IVW, inverse variance weighted; MR, Mendelian randomization; NA, not available; SE, standard error.

Table S10. MVMR estimating the association of each mediator with PPD with adjustment for education attainment

<table>
<tr><th rowspan="2">Mediator</th><th rowspan="2">Method</th><th rowspan="2">Variable</th><th rowspan="2">β</th><th rowspan="2">SE</th><th rowspan="2">P</th><th colspan="3">MVMR
Instrument validity test</th><th colspan="2">MVMR
Heterogeneity</th><th colspan="2">MVMR
Pleiotropy test</th></tr>
<tr><th>F statistic</th><th>Q statistic</th><th>P</th><th>Q statistic</th><th>P</th><th>Egger intercept</th><th>P</th></tr>
<tr><td rowspan="4">Age at first live birth</td><td rowspan="2">MV-IVW</td><td>Years of schooling</td><td>0.19</td><td>0.31</td><td>0.531</td><td rowspan="4">13.22</td><td rowspan="4">92.78</td><td rowspan="4">6.95E-06</td><td rowspan="2">98.65</td><td rowspan="2">< 0.05</td><td rowspan="4">0.005</td><td rowspan="4">0.60</td></tr>
<tr><td>Age at first live birth</td><td>-1.00</td><td>0.26</td><td>9.00E-05</td></tr>
<tr><td rowspan="2">MVMR Egger</td><td>Years of schooling</td><td>0.05</td><td>0.41</td><td>0.906</td><td rowspan="2">98.01</td><td rowspan="2">< 0.05</td></tr>
<tr><td>Age at first live birth</td><td>-1.11</td><td>0.34</td><td>0.001</td></tr>
<tr><td rowspan="4">Neuroticism score</td><td rowspan="2">MV-IVW</td><td>Years of schooling</td><td>-0.45</td><td>0.17</td><td>7.56E-03</td><td rowspan="4">17.53</td><td rowspan="4">163.65</td><td rowspan="4">0.001</td><td rowspan="2">166.83</td><td rowspan="2">< 0.05</td><td rowspan="4">0.004</td><td rowspan="4">0.08</td></tr>
<tr><td>Neuroticism score</td><td>0.15</td><td>0.04</td><td>4.53E-05</td></tr>
<tr><td rowspan="2">MVMR Egger</td><td>Years of schooling</td><td>-0.71</td><td>0.22</td><td>0.001</td><td rowspan="2">155.42</td><td rowspan="2">< 0.05</td></tr>
<tr><td>Neuroticism score</td><td>0.16</td><td>0.04</td><td>0.000</td></tr>
<tr><td rowspan="4">Average total household income before tax</td><td rowspan="2">MV-IVW</td><td>Years of schooling</td><td>-0.34</td><td>0.33</td><td>0.314</td><td rowspan="4">3.07</td><td rowspan="4">87.81</td><td rowspan="4">0.0001</td><td rowspan="2">88.38</td><td rowspan="2">< 0.05</td><td rowspan="4">0.013</td><td rowspan="4">0.30</td></tr>
<tr><td>Average total household income before tax</td><td>-0.14</td><td>0.31</td><td>0.668</td></tr>
<tr><td rowspan="2">MVMR Egger</td><td>Years of schooling</td><td>-0.60</td><td>0.42</td><td>0.154</td><td rowspan="2">86.35</td><td rowspan="2">< 0.05</td></tr>
<tr><td>Average total household income before tax</td><td>-0.65</td><td>0.59</td><td>0.271</td></tr>
</table>

Abbreviations: MV-IVW, multivariable inverse variance weighted; MVMR, multivariable Mendelian randomization; SE, standard error.
